# Hairy Cell Leukemia and Its Mimics: A Case-Based Exploration of Diagnostic Complexity

**DOI:** 10.7759/cureus.83024

**Published:** 2025-04-26

**Authors:** Omar A Al Lehyani, Mazen Al Malki, Rawiah A AlAmmary, Nojood A Althubaity, Muhammad Kashif, Muhammad A Qureshi, Hamad M AlGethami, Rasha Mutabaqani, Salwa H Alhassani, Ahmed Embaby, Noha Elnagdy, Ahmed K Shawush, Abdullah S Alsulaiman

**Affiliations:** 1 Medical Laboratory, Al Hada Armed Forces Hospital, Taif, SAU; 2 Hematology and Oncology Department, Al Hada Armed Forces Hospital, Taif, SAU; 3 Medical Laboratory, King Abdullah Medical City, Mecca, SAU

**Keywords:** bone marrow fibrosis, braf v600e mutation, cd103 expression, cd200 marker, flow cytometry, hairy cell leukemia, hcl variant, immunophenotype, molecular diagnostics, monocytopenia

## Abstract

Hairy cell leukemia (HCL) is a rare but well-characterized B-cell malignancy with distinct immunophenotypic and genetic features. Advances in molecular diagnostics, particularly the identification of the BRAF V600E mutation, have significantly enhanced diagnostic precision and informed targeted treatment strategies. However, the HCL variant and other HCL-like syndromes continue to present substantial diagnostic challenges due to their absence of BRAF mutations, atypical immunophenotypic profiles, and divergent clinical courses. This case report provides an overview of the morphological, immunophenotypic, molecular, and histopathological features of HCL, as well as its differential diagnoses, with a focus on the utility of flow cytometry and emerging molecular diagnostic markers. To underscore the practical implications of these diagnostic complexities, we present a case report that reinforces the challenges in distinguishing HCL from its mimics and highlights the role of contemporary integrated diagnostic methodologies.

## Introduction

Hairy cell leukemia (HCL) is a rare, chronic B-cell lymphoproliferative disorder first described in 1958. It accounts for approximately 2% of all leukemias, with an estimated incidence of 0.3 cases per 100,000 individuals per year [1]. HCL predominantly affects males, with a male-to-female ratio of approximately 4:1, and typically presents in individuals between the ages of 50 and 60 years [2]. Although its etiology remains incompletely understood, a pivotal discovery has been its strong association with the BRAF V600E mutation, which is now recognized as a central driver in disease pathogenesis [3,4].

Clinically, HCL often presents insidiously with nonspecific symptoms, such as fatigue, recurrent infections, and abdominal discomfort, manifestations that can be misattributed to more common conditions. Hematologically, it is characterized by pancytopenia, monocytopenia, and splenomegaly, with typically absent lymphadenopathy, a feature that helps distinguish it from other indolent B-cell malignancies [4].

However, accurate diagnosis remains a challenge, particularly in distinguishing classic HCL from its close mimics: HCL variant (HCL-v), splenic marginal zone lymphoma (SMZL), and splenic diffuse red pulp lymphoma (SDRPL). These entities may share overlapping clinical and morphological features, especially in the early stages of the disease, which can complicate diagnostic precision [5,6].

In the fifth edition of the WHO Classification of Haematolymphoid Tumours (2022), HCL is categorized under splenic B-cell lymphomas and leukemias, a subgroup of mature B-cell neoplasms. HCL remains a distinct entity based on its unique morphology, immunophenotype, and the BRAF V600E mutation. The updated classification also introduces splenic B-cell lymphoma/leukemia with prominent nucleoli (SBLPN) to replace the term “hairy cell leukemia variant,” recognizing its distinct biology. Other entities in this group include SMZL and splenic diffuse red pulp small B-cell lymphoma [6].

Classic HCL is defined immunophenotypically by the co-expression of CD11c, CD25, CD103, and CD123 and harbors the BRAF V600E mutation in over 90% of cases, a molecular hallmark critical for diagnosis confirmation. In contrast, HCL-v lacks expression of CD25 and CD123, does not carry the BRAF BRAF V600E mutation, and typically presents with higher leukocyte counts and poorer response to standard purine analog therapy. SMZL, while similarly involving splenomegaly and cytopenias, is characterized by circulating villous lymphocytes and lacks both the BRAF BRAF V600E mutation and the immunophenotypic profile of HCL.

Misclassification of HCL has significant clinical consequences. Administering purine analogs to patients with SBLPN, where efficacy is limited, or delaying appropriate therapy in classic HCL can result in prolonged cytopenias, recurrent infections, drug-related toxicities, disease progression, and missed opportunities for targeted interventions such as BRAF inhibitors. These risks highlight the importance of a multidisciplinary diagnostic approach, which integrates cytomorphology, flow cytometry, immunohistochemistry, and molecular testing, to achieve diagnostic accuracy and inform optimal treatment strategies [7].

This article provides a comprehensive overview of the diagnostic framework for HCL, emphasizing the clinical, morphological, immunophenotypic, and molecular characteristics that distinguish it from related splenic B-cell neoplasms. Through a detailed, case-based analysis, we aim to demonstrate how an integrated diagnostic approach, incorporating flow cytometry, immunohistochemistry, and molecular assays, can overcome diagnostic challenges, ensure accurate classification, and inform evidence-based treatment decisions in patients with suspected HCL or its mimics.

## Case presentation

A 64-year-old male presented with progressive thrombocytopenia, which subsequently evolved into pancytopenia. He had no prior history of hematologic disorders or significant infections. Physical examination revealed mild splenomegaly without evidence of lymphadenopathy. The patient reported two previous episodes of COVID-19 infection, neither of which required hospitalization. Abdominal ultrasound demonstrated a mildly enlarged spleen measuring 15.6 cm, with no focal lesions identified.

The patient's lab results revealed mild pancytopenia, characterized by a decreased white blood cell count, as well as reduced counts of neutrophils, monocytes, and platelets. Hemoglobin levels remain within normal limits, indicating that red cell production is preserved. A reticulocyte count was performed and found to be inappropriately low in the context of anemia, indicating hypoproliferative marrow activity rather than peripheral loss or destruction. Notably, the absolute counts of neutrophils and monocytes are significantly reduced, suggesting potential bone marrow suppression. Additionally, the serum vitamin B12 level was normal, which, in the absence of hypersegmented neutrophils on peripheral smear, excluded megaloblastic anemia as a contributing factor. Despite these abnormalities, lactate dehydrogenase (LDH) levels are normal, indicating that there is no overt cell lysis or a high tumor burden (Table 1).

**Table 1:** Laboratory findings

Parameter	Patient's value	Reference range
Hemoglobin	14.3 g/dL	12.0-16.0 g/dL
White blood cell count	↓ 3.71 × 10⁹/L	4.0-11.0 × 10⁹/L
Neutrophils	↓ 0.9 × 10⁹/L	2.0-7.5 × 10⁹/L
Lymphocytes	3.42 × 10⁹/L	1.0-4.4 × 10⁹/L
Monocytes	↓ 0.16 × 10⁹/L	0.2-1.0 × 10⁹/L
Platelets	↓ 97 × 10⁹/L	150-450 × 10⁹/L
Reticulocyte count	↓ 29 × 10⁹/L	30-137 × 10⁹/L
Vitamin B12	850 pg/mL	200-900 pg/mL
Lactate dehydrogenase	145	135 – 243 U/L

Peripheral blood smear and bone marrow morphology

**Figure 1:**
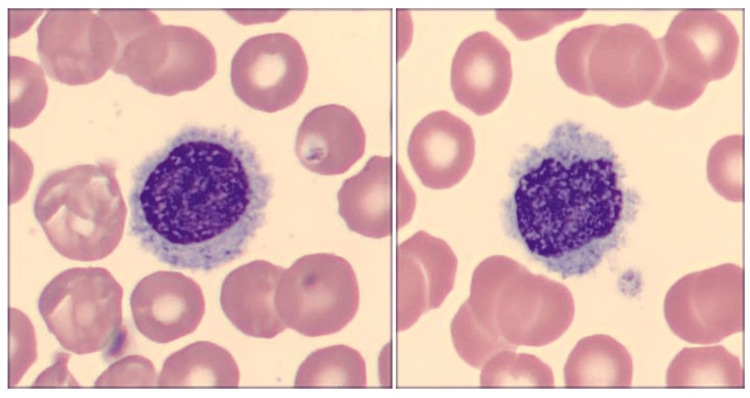
Morphological features of hairy cell in peripheral blood Patient's peripheral blood smears at x1000 magnification display classic hairy cells with round to oval nuclei, loose chromatin, and irregular cytoplasmic projections.

**Figure 2:**
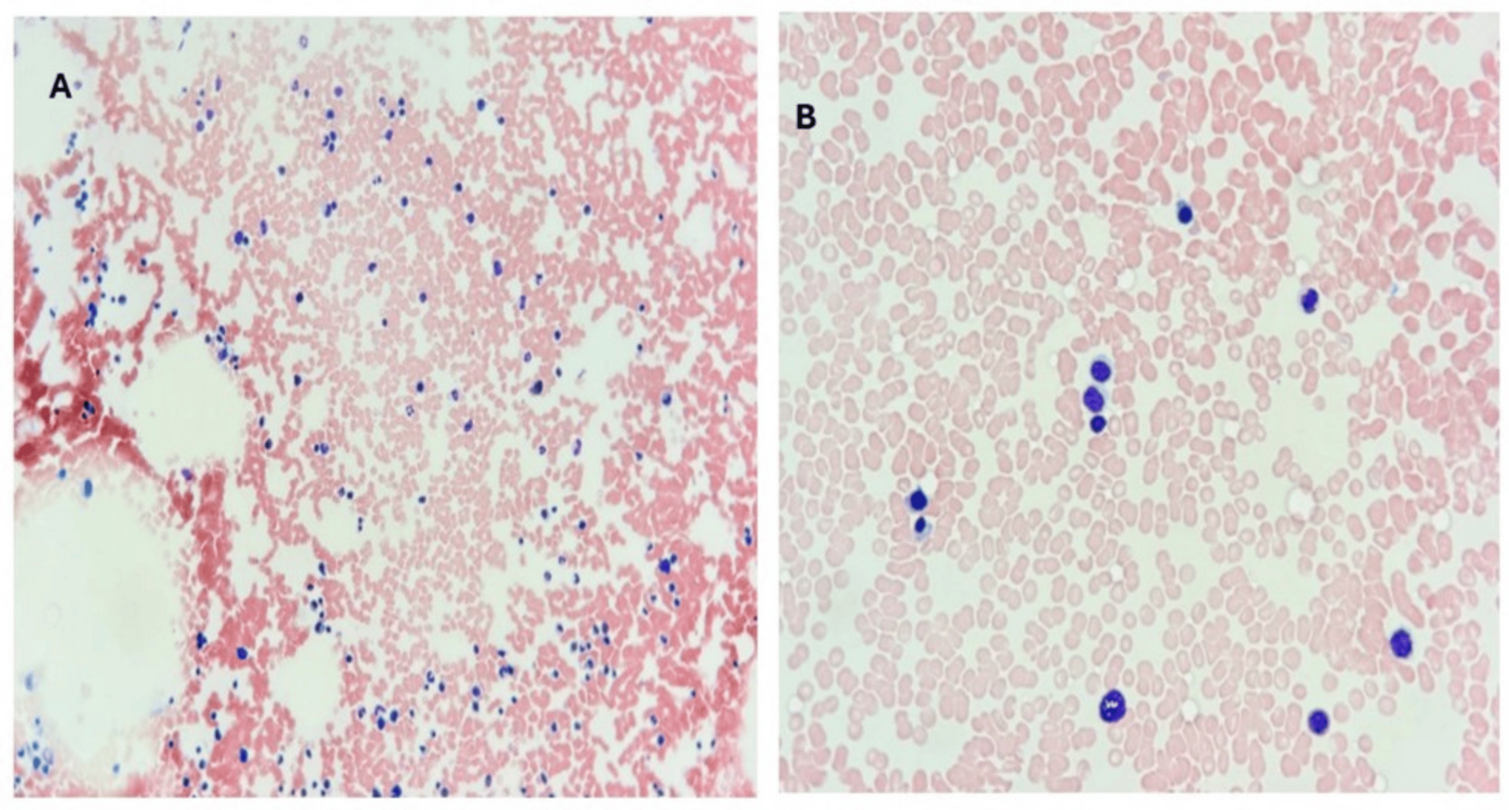
Bone marrow aspirate in HCL Patient's bone marrow smear. (A) At x200, hypocellular marrow with diffuse infiltration by small- to medium-sized lymphoid cells exhibits features consistent with hairy cells. (B) The same marrow aspiration at x400 highlights individual hairy cells with oval nuclei and pale, moderately abundant cytoplasm. HCL: hairy cell leukemia

**Figure 3:**
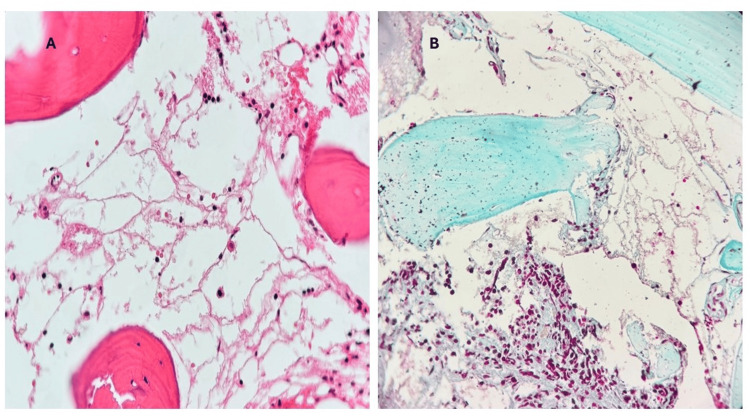
Bone marrow trephine biopsy (A) H&E stain at x400 demonstrates a markedly hypocellular marrow with widespread fatty replacement, consistent with the marrow suppression seen in HCL. (B) Masson’s trichrome stain at x200 revealed fibrotic changes, evidenced by blue-stained collagen fibers replacing normal hematopoietic elements, indicating the onset of reticulin fibrosis associated with disease progression. A reticulin stain (not shown) demonstrated grade 2 reticulin fibrosis, according to the WHO grading system, supporting the diagnosis of classic HCL. These histological features are characteristic of HCL and contribute to the observed pancytopenia. H&E: hematoxylin and eosin, HCL: hairy cell leukemia, WHO: World Health Organization

Peripheral blood smear revealed scattered small lymphoid cells with abundant pale cytoplasm and cytoplasmic projections ("hairy cells") (Figure 1). A bone marrow trephine biopsy and aspirate were performed. The aspirate was hypocellular (Figure 2). The trephine biopsy revealed a markedly hypocellular marrow with interstitial infiltration by small lymphoid cells, characterized by oval nuclei and abundant cytoplasm (Figure 3).

Flow cytometry findings

**Figure 4:**
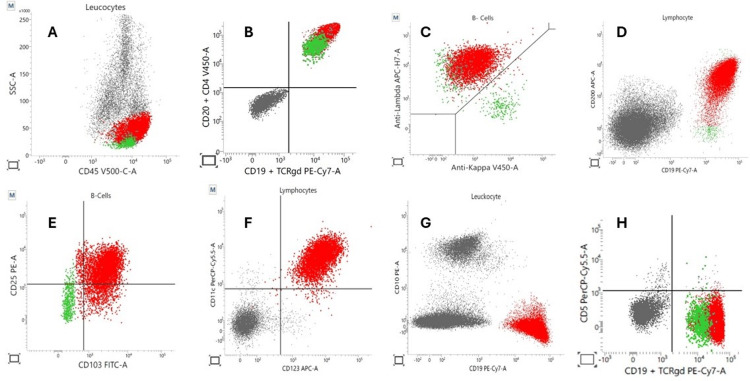
Flow cytometric immunophenotyping Panels A–H illustrate multiparametric flow cytometric analysis of our patient's peripheral blood samples. The red population represents malignant HCL cells, and the green population corresponds to residual normal B-cells. Gray represents non-B-cell events (e.g., T-cells, NK cells, or background). (A) CD45 vs. SSC: The HCL cells (red) exhibit increased SSC due to their cytoplasmic projections, a hallmark of hairy cells. This elevated SSC may cause these cells to fall outside the typical lymphocyte gate if not carefully analyzed. (B) CD19 vs. CD20: HCL cells co-express CD19 and brightly express CD20, consistent with a mature B-cell phenotype. (C) The HCL population shows surface light chain restriction (in this case, IgK), confirming monoclonality. (D) CD19 vs. CD200: HCL cells express CD200 brightly, in contrast to the dimmer or absent expression on normal B-cells. (E) CD103 and CD25: Two highly characteristic markers for HCL, distinguishing HCL from HCL-v, which lacked CD25 expression. (F) CD11c vs. CD123: Strong co-expression of CD11c and CD123 is another diagnostic hallmark of HCL. (G) CD19 vs. CD10: Showing that HCL lacks CD10 expression. (H) CD19 vs. CD5: Showing that HCL lacks CD5 expression HCL: hairy cell leukemia, SSC: side scatter, HCL-v: hairy cell leukemia variant

**Figure 5:**
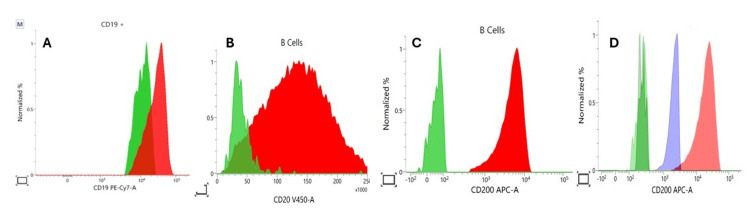
Flow cytometry histograms illustrating the intensity of expression in HCL (red) compared to normal B-cells (green) and CLL (blue) Panels A–D display normalized fluorescence intensity for selected markers on gated CD19⁺ B-cells. The red population represents malignant HCL cells, the green population represents residual normal B-cells, and the blue population in Panel D represents CLL cells. (A, B, C) CD19 and CD20 expression is overexpressed in HCL cells relative to normal B-cells. (D) Comparative CD200 expression highlights its diagnostic value: HCL (red) shows markedly higher expression than CLL cells (blue) and normal B-cells (green), supporting its utility in distinguishing HCL from other B-cell lymphoproliferative disorders. HCL: hairy cell leukemia, CLL: chronic lymphocytic leukemia The instrument used for this analysis is BD FACSLyric^TM ^(BD Biosciences, Franklin Lakes, NJ, USA), and the software used for data analysis is BD FACSSuite^TM ^(BD Biosciences, Franklin Lakes, NJ, USA).

Flow cytometry of peripheral blood demonstrated a monoclonal B-cell population positive for CD19, CD20 (bright), CD22, CD11c, CD103, CD123, CD25, and bright expression of CD200 and negative for CD5, CD10, and CD23 (Figures 4-5).

Molecular testing and final diagnosis

Given the high clinical suspicion for HCL, molecular testing for the BRAF V600E mutation was performed using a polymerase chain reaction (PCR)-based assay, which confirmed its presence and thereby supported the diagnosis of classic HCL. The differential diagnosis included HCL-v and SMZL. However, detecting the BRAF V600E mutation and strong expression of CD25, CD123, and CD200 (bright) further favored classic HCL over its mimics.

Beyond its diagnostic significance, identifying the BRAF V600E mutation in this patient carries important therapeutic and prognostic implications. It serves as a predictive biomarker for response to BRAF inhibitor therapy. It has demonstrated substantial efficacy in cases of relapsed or refractory HCL, particularly when first-line purine analog treatment is unsuccessful. In addition, the presence of the mutation informs post-treatment monitoring strategies, enabling the use of molecular minimal residual disease (MRD) assessment via highly sensitive PCR or next-generation sequencing. These approaches can guide the timing and intensity of follow-up interventions, supporting a more tailored and responsive management plan.

Treatment and post-diagnosis management

The patient was started on standard first-line therapy with cladribine at a dose of 0.15 mg/kg/day, administered intravenously over two hours for five consecutive days. This was followed by rituximab, administered weekly for eight weeks, starting one month after the completion of cladribine, in line with current evidence showing improved remission rates with this combination.

To prevent opportunistic infections during and after treatment, prophylactic antimicrobial therapy was initiated, including valacyclovir and trimethoprim-sulfamethoxazole (Bactrim), to be continued for a minimum of six months. Then, a bone marrow evaluation is scheduled six months after therapy, including flow cytometric analysis for MRD and molecular testing for the BRAF V600E mutation, to assess the depth of response and guide future management decisions.

In the event of early relapse, the treatment plan includes the consideration of a selective BRAF inhibitor, reflecting current therapeutic advancements in the management of relapsed or refractory HCL.

## Discussion

HCL is a rare but well-characterized B-cell malignancy with a unique clinical, morphological, immunophenotypic, and molecular profile. The disease typically affects older adults, most often males, and although it follows an indolent course, it can lead to significant clinical morbidity. Its presentation commonly includes cytopenias, splenomegaly, and frequent diagnostic challenges that require a high index of clinical suspicion. This case-based review provides insight into how individual clinical, laboratory, and molecular features gather to guide diagnosis, using our patient’s course as a framework to highlight key diagnostic principles.

In the presented case, a 64-year-old male exhibited mild splenomegaly without lymphadenopathy and initially presented with progressive thrombocytopenia, which subsequently evolved into pancytopenia. This clinical course is characteristic of classic HCL, particularly when hemoglobin levels are preserved and there is a marked reduction in monocyte and neutrophil counts. The absence of lymphadenopathy is a typical finding in HCL, reflecting the disease’s preferential infiltration of the bone marrow, spleen, and peripheral blood rather than lymph nodes. This distinctive tissue tropism is partly mediated by the increased expression of adhesion molecules, such as CD11c and CD103, on hairy cells, which facilitates their retention within the splenic red pulp and the bone marrow stroma. As a result, splenomegaly and marrow fibrosis are commonly observed, whereas lymph node involvement remains rare. In our patient, the lack of overt infections despite profound cytopenias underscores the indolent yet immunosuppressive nature of HCL. Furthermore, LDH levels remained within the normal range, which reinforces the disease’s low proliferative index and helps distinguish it from more aggressive lymphoid neoplasms [8].

The hallmark laboratory feature in HCL is pancytopenia, particularly monocytopenia, which is uncommon in other chronic B-cell malignancies. Our patient’s absolute monocytopenia and neutropenia provided an early indication of possible marrow infiltration. In HCL, the marrow becomes increasingly fibrotic and hypocellular due to cytokine-mediated interactions between leukemic cells and the stromal microenvironment [9]. This fibrosis contributes to the typical “dry tap” during bone marrow aspiration and necessitates a biopsy to establish a definitive diagnosis, as was the case in our patient.

Morphologically, peripheral blood and bone marrow findings further supported HCL in our patient. The characteristic “hairy” cytoplasmic projections on lymphocytes, visible in peripheral smears, along with interstitial marrow infiltration by small B cells with oval nuclei and abundant cytoplasm, remain key diagnostic clues [10,11]. These findings are not only pathognomonic but also help differentiate HCL from mimickers, such as HCL-v and SMZL, which lack these cytological features.

Flow cytometry remains the cornerstone of HCL diagnosis. Our patient's immunophenotypic profile (strong expression of CD11c, CD25, CD103, and CD123, and bright CD200, with the absence of CD5, CD10, and CD23) was diagnostic. These markers are unique to classic HCL and seldom co-expressed in other B-cell malignancies. Table 2 outlines the comparative expression of key markers among HCL, HCL-v, and SMZL, and how these guide diagnostic clarity. Notably, CD200 is a specific marker that is brightly expressed only in classic HCL. This was evident in our patient, where CD200 expression was more intense than in chronic lymphocytic leukemia (CLL), helping reinforce the distinction. CD123 is another discriminator, being positive only in HCL and absent in HCL-v and SMZL. Its detection in our patient supported the diagnosis of classic HCL. CD103 expression overlaps between HCL and HCL-v but is not seen in SMZL. Thus, co-expression with CD123 and CD25 further enhances diagnostic specificity. The co-expression of CD25, CD123, and CD103, as seen in our case, forms a reliable immunophenotypic signature that favors classic HCL over its mimics [10,11].

**Table 2:** Key immunophenotypic markers in HCL and its variants HCL: hairy cell leukemia, HCL-v: hairy cell leukemia variant, SMZL: splenic marginal zone lymphoma

Marker	HCL	HCL-v	SMZL
CD19/CD20 [2,3]	+	+	+
CD11c [3,10]	+ (bright)	+ (dim)	-
CD103 [3,10]	+	+	-
CD25 [2,3,6]	+	-	-
CD123 [3,4,10]	+	-	-
CD200 [3,4,10]	+ (bright)	-	-
Annexin A1 [4,5]	+	-	-

The definitive diagnostic step was molecular confirmation of the BRAF V600E mutation. This mutation, present in nearly all cases of classic HCL, drives the constitutive activation of the MAPK/ERK pathway, promoting cell survival and proliferation while inhibiting apoptosis [5,12]. Detection of this mutation ruled out HCL-v, which lacks the BRAF V600E mutation, and helped distinguish the disease from morphologically similar conditions. Our patient's mutation profile was consistent with classic HCL and aligned with the immunophenotypic and histologic findings.

Table 3 focuses on the pathophysiology of the various immunophenotypic markers. A detailed understanding of the markers in this table offers insights into the disease’s biology and aids in precise classification. CD25 and CD123 both contribute to the immunologic microenvironment that supports the survival of leukemic cells. CD25, the alpha chain of the IL-2 receptor, may enhance the responsiveness of leukemic cells to T-cell-derived growth signals. In contrast, CD123, the alpha chain of the IL-3 receptor, promotes survival and proliferation through cytokine-mediated pathways. Annexin A1, a particular marker of classic HCL, inhibits apoptosis by reinforcing the BRAF-MEK-ERK signaling axis and is not found in HCL-v. Its immunohistochemical detection is particularly valuable in cases with dry tap bone marrows. The presence of the BRAF V600E mutation exclusively in classic HCL has both diagnostic and therapeutic implications; it confirms the diagnosis molecularly and makes patients eligible for BRAF inhibitor therapies. Clinically, these differences translate into a more indolent course for classic HCL compared to the often aggressive and treatment-resistant HCL-v. Table 3 supports and emphasizes the critical interplay between surface immunophenotype, underlying signaling biology, and clinical behavior.

**Table 3:** Key immunophenotypic markers in HCL This table summarizes the key immunophenotypic markers in HCL and their roles in disease pathogenesis and clinical diagnosis. Each marker is associated with specific functions that contribute to tumor progression, immune evasion, and bone marrow retention, which in turn have critical diagnostic and therapeutic implications. Content synthesized from references [3-5,10,13] regarding immunophenotype and functional relevance. HCL: hairy cell leukemia

Marker	Function in HCL pathogenesis	Clinical and diagnostic impact
CD11c	Enhances interaction with the bone marrow stromal environment [3,10]	Contributes to tumor survival
CD103	Mediates adhesion to stromal fibroblasts and endothelial cells [3,10]	Promotes tissue infiltration (spleen, bone marrow)
CD25 (IL-2 receptor α-chain)	Enhance responsiveness to growth signals, sustaining proliferation [3,4,10]	Aids in flow cytometry diagnosis
CD123 (IL-3 receptor α-chain)	Enhances cytokine-mediated survival [3,4,13]	Higher expression differentiates classic HCL from HCL-v
CD200	Immune evasion via inhibition of macrophage activity [3,4,5]	Correlates with indolent disease
Annexin A1	Regulates apoptosis resistance via BRAF-MEK signaling [5,12]	A marker that distinguishes HCL from HCL-v

Table 4 illustrates the key pathophysiologic mechanisms underlying HCL, along with their corresponding clinical manifestations and diagnostic findings. For instance, BRAF V600E-driven MAPK pathway activation leads to cytopenias, including monocytopenia, which are often accompanied by neutropenia, anemia, and thrombocytopenia. Splenic infiltration by HCL cells results in splenomegaly and abdominal discomfort, which can be detected via imaging. Bone marrow fibrosis, often cytokine-mediated, causes the classic "dry tap" on aspiration, confirmed histologically through reticulin deposition. CXCR4-mediated leukemic cell retention within the marrow and splenic niches contributes to disease persistence and therapeutic resistance, which can be assessed through MRD monitoring using flow cytometry. Each mechanism connects a biologic alteration with a clinical consequence, helping guide accurate diagnosis and tailored treatment.

**Table 4:** Key pathophysiologic mechanisms underlying HCL This table illustrates the key pathophysiologic mechanisms underlying HCL, along with their corresponding clinical manifestations and diagnostic findings. Each biological alteration contributes to specific disease characteristics that help differentiate HCL from other B-cell malignancies and guide appropriate diagnostic and therapeutic approaches. HCL: hairy cell leukemia This table is based on clinical insights supported by references [2-5,7,8,13] regarding disease biology and corresponding clinical findings.

Pathophysiologic feature	Clinical manifestation	Diagnostic finding
BRAF V600E-driven proliferation [4,5,12]	Cytopenias, monocytopenia	Neutropenia, anemia, thrombocytopenia
Splenic infiltration by HCL cells [2,6,7]	Splenomegaly, abdominal discomfort	Massive spleen enlargement on imaging
Bone marrow fibrosis [3,8,10]	"Dry tap" on aspiration	Reticulin deposition on biopsy
CXCR4-mediated microenvironment retention [5,8,13]	Persistent disease, drug resistance	MRD detection via flow cytometry

While the diagnosis of HCL may appear straightforward when classic features are present, the disease is frequently underrecognized, particularly in its early stages. Delays in diagnosis can result in persistent cytopenias, infectious complications, and inappropriate therapeutic strategies. In our case, the timely recognition of the diagnostic pattern led to early confirmation and enabled the consideration of appropriate treatment strategies. Clinicians must be vigilant when encountering patients with unexplained pancytopenia and splenomegaly, even in the absence of lymphadenopathy or systemic symptoms.

With the advent of targeted therapies such as BRAF inhibitors, the outlook for HCL has improved considerably. However, challenges persist, especially in detecting MRD, managing relapsed cases, and addressing bone marrow fibrosis and immune dysfunction. Our case illustrates the importance of synthesizing clinical and laboratory data in a structured diagnostic pathway to arrive at a timely and accurate diagnosis. Early diagnosis reduces morbidity and ensures the use of disease-specific therapies, improving long-term outcomes.

## Conclusions

HCL and its variants pose notable diagnostic challenges due to overlapping morphological and immunophenotypic features with other indolent B-cell neoplasms. This case-based review highlights the essential role of an integrated diagnostic approach encompassing clinical evaluation, peripheral blood smear analysis, flow cytometry, and molecular testing. The identification of hallmark immunophenotypic markers, such as CD25, CD103, CD200, and Annexin A1, together with the detection of the BRAF V600E mutation, is crucial for the accurate diagnosis of classic HCL and its differentiation from mimics, including HCL-v, SMZL, and SDRPL. Early and precise diagnosis enables the timely initiation of targeted therapies and enhances the monitoring of MRD, contributing to improved patient outcomes.

In patients presenting with unexplained cytopenias, splenomegaly, and absence of lymphadenopathy, clinicians should maintain a high index of suspicion for HCL. Early use of flow cytometry and molecular testing, particularly for the BRAF V600E mutation, can facilitate prompt and accurate diagnosis, allowing for the timely initiation of targeted therapy and improving prognosis and long-term disease control.
